# Supplementary material for: Experimental validation of otolith-based age and growth reconstructions across multiple life stages of a critically endangered estuarine fish
Source: PeerJ. 2021 Nov 17;9:e12280. doi: 10.7717/peerj.12280 (PMC8605759; doi:10.7717/peerj.12280)
Supplement: Supplemental Information 1 — Length conversion equations for larval and adult Delta Smelt measured fresh by hand or digitally using image analysis (A–B), preserved in ethanol (C–D) or frozen at −20 °C (E–F), and fork length (FL) conversions from SL and TL of frozen specimens. Dashed lines represent 1:1; red lines represent the respective linear models. Linear models and R2 values are proved in the top-left of each plot; proportional adjustment functions are provided in the bottom-right (C–H). [file peerj-09-12280-s001.pdf]

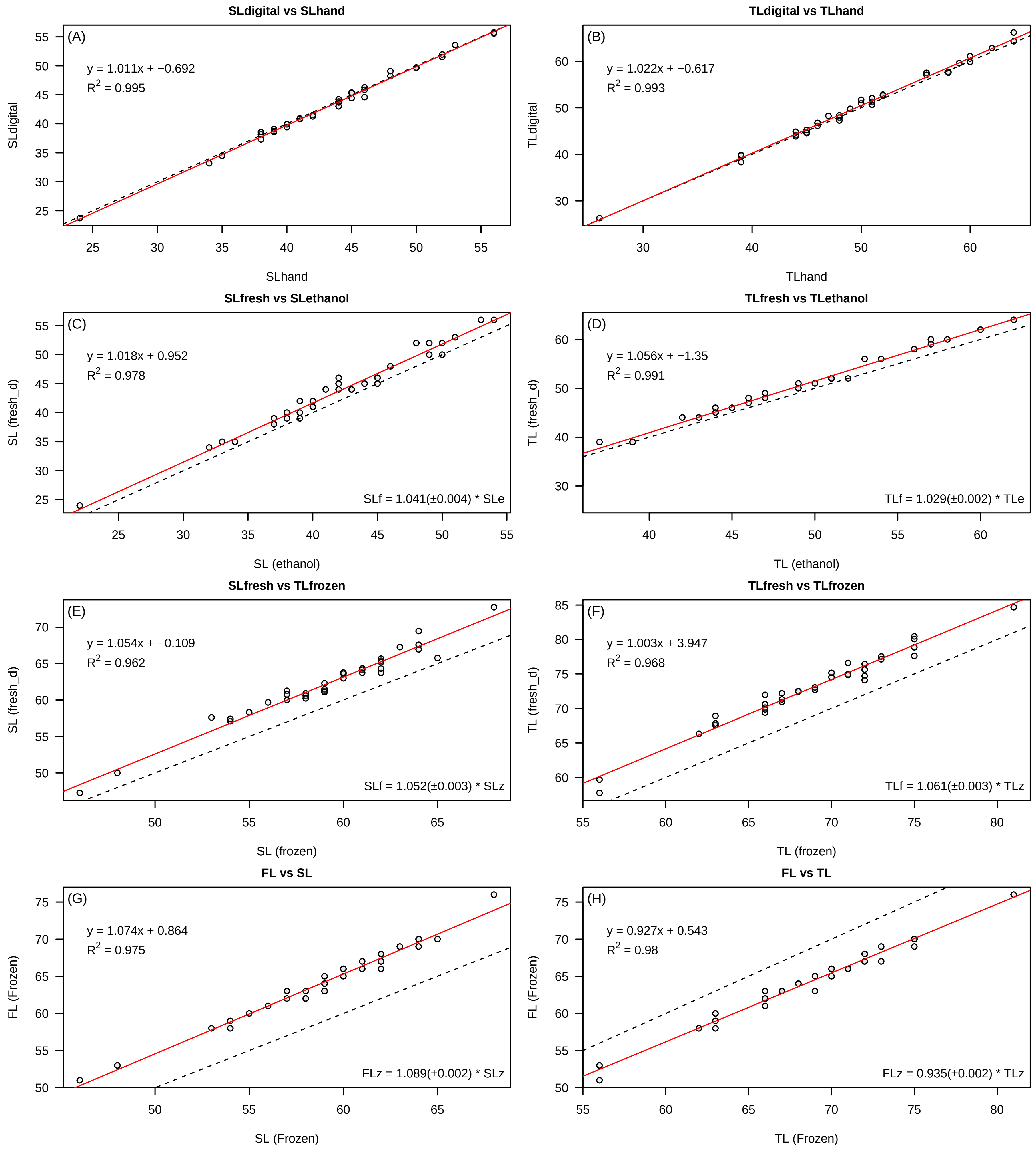
SLdigital vs SLhand
(A)
y = 1.011x + −0.692
R^2 = 0.995
SLdigital
SLhand
TLdigital vs TLhand
(B)
y = 1.022x + −0.617
R^2 = 0.993
TLdigital
TLhand
SLfresh vs SLethanol
(C)
y = 1.018x + 0.952
R^2 = 0.978
SLf = 1.041(±0.004) * SLe
SL (fresh_d)
SL (ethanol)
TLfresh vs TLethanol
(D)
y = 1.056x + −1.35
R^2 = 0.991
TLf = 1.029(±0.002) * TLe
TL (fresh_d)
TL (ethanol)
SLfresh vs TLfrozen
(E)
y = 1.054x + −0.109
R^2 = 0.962
SLf = 1.052(±0.003) * SLz
SL (fresh_d)
SL (frozen)
TLfresh vs TLfrozen
(F)
y = 1.003x + 3.947
R^2 = 0.968
TLf = 1.061(±0.003) * TLz
TL (fresh_d)
TL (frozen)
FL vs SL
(G)
y = 1.074x + 0.864
R^2 = 0.975
FLz = 1.089(±0.002) * SLz
FL (Frozen)
SL (Frozen)
FL vs TL
(H)
y = 0.927x + 0.543
R^2 = 0.98
FLz = 0.935(±0.002) * TLz
FL (Frozen)
TL (Frozen)
