# Supplementary figures and images for: Experimental validation of otolith-based age and growth reconstructions across multiple life stages of a critically endangered estuarine fish
Source: PeerJ. 2021 Nov 17;9:e12280. doi: 10.7717/peerj.12280 (PMC8605759; doi:10.7717/peerj.12280)

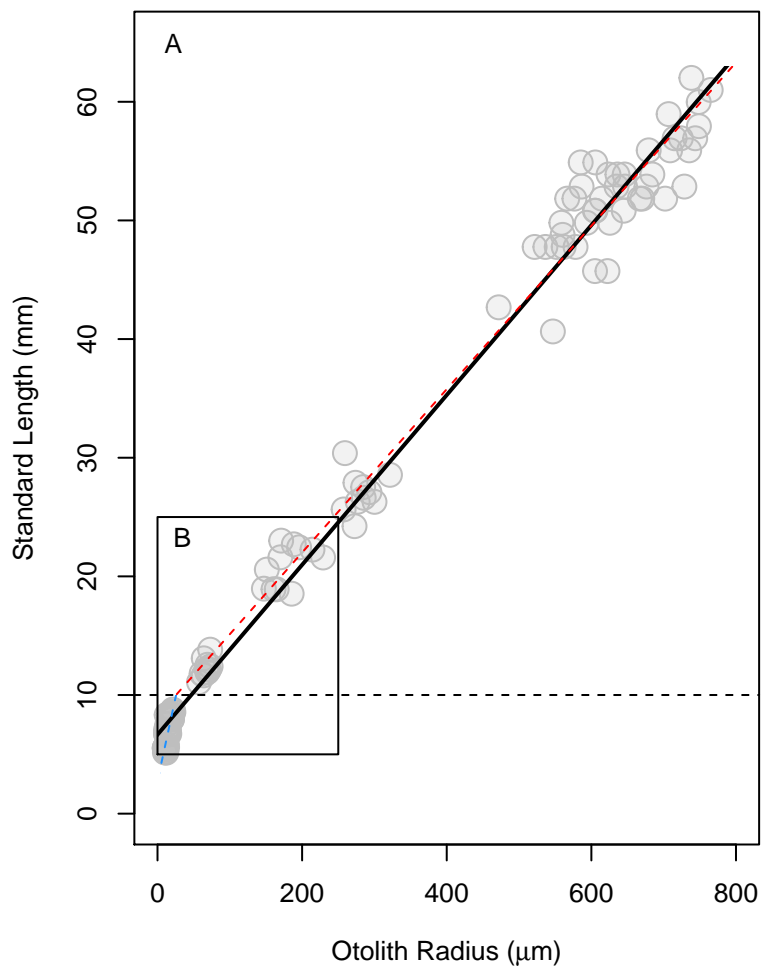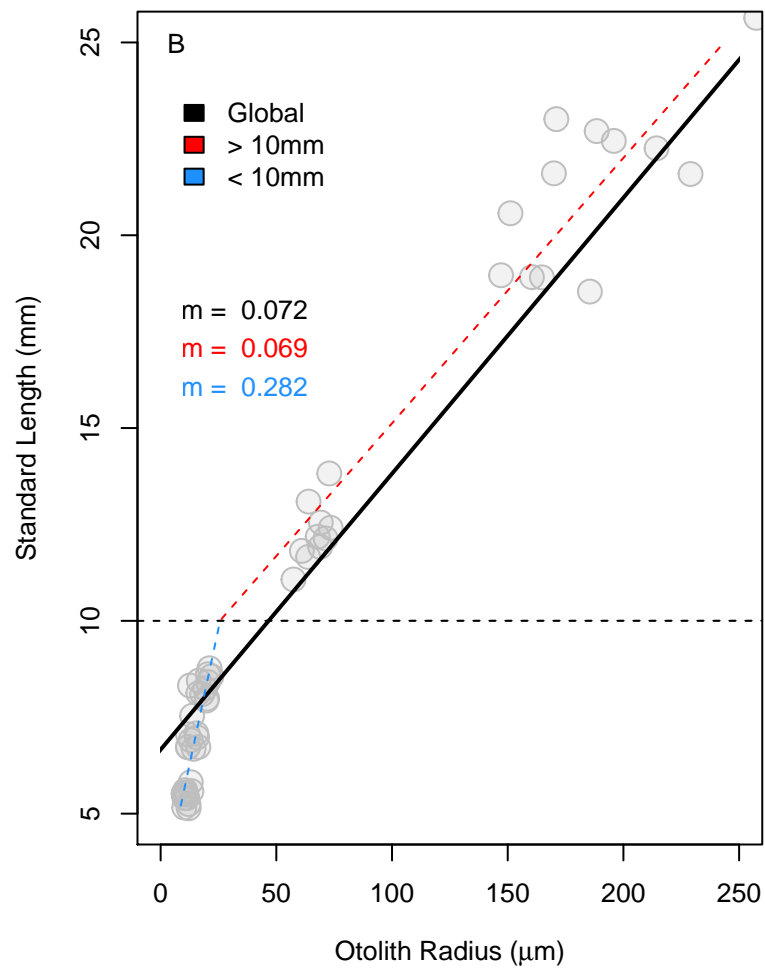

Supplement: Supplemental Information 2 — Plots showing all size classes (A) and fish < 25 mm (B) are provided. Three models were fit including a global model (all size classes as in Fig. 1B, black), a model for pro-larval fish ≤ 10 mm (blue), and a model for all fish > 10 mm (red). Slopes (m) of each model are provided in (b) (see Table S3 for model details). [file peerj-09-12280-s002.pdf]
