# Supplementary material for: Experimental validation of otolith-based age and growth reconstructions across multiple life stages of a critically endangered estuarine fish
Source: PeerJ. 2021 Nov 17;9:e12280. doi: 10.7717/peerj.12280 (PMC8605759; doi:10.7717/peerj.12280)
Supplement: Supplemental Information 3 — Table headings include: Model = fish size class, Coefficient = slope or intercept for each linear model, Estimate = coefficient value, Error = standard error, t = t-value, P1 = p-value for each coefficient, n = sample size, F = F-statistic, P2 = p-value and R2 = coefficient of determination for the model. SL = standard length. [file peerj-09-12280-s003.docx]

| **Model** | **Coefficient** | **Estimate** | **Error** | **t** | **P_1_** | **n** | **F** | **P_2_** | **R^2^** |
| --- | --- | --- | --- | --- | --- | --- | --- | --- | --- |
| All Sizes | Intercept | 7.830 | 0.316 | 24.770 | < 0.001 | 108 | 8694.2 | < 0.001 | 0.989 |
|  | Slope | 0.070 | 0.001 | 93.240 | < 0.001 |  |  |  |  |
| > 10 mm SL | Intercept | 8.997 | 0.576 | 15.620 | < 0.001 | 76 | 3508.1 | < 0.001 | 0.979 |
|  | Slope | 0.068 | 0.001 | 59.230 | < 0.001 |  |  |  |  |
| ≤ 10 mm SL | Intercept | 4.053 | 0.544 | 7.449 | < 0.001 | 32 | 61.1 | < 0.001 | 0.671 |
|  | Slope | 0.285 | 0.036 | 7.817 | < 0.001 |  |  |  |  |
